# Effectiveness of mechanical and chemical decontamination methods for the treatment of dental implant surfaces affected by peri‐implantitis: A systematic review and meta‐analysis

**DOI:** 10.1002/cre2.839

**Published:** 2024-02-06

**Authors:** Iain Hart, Christine Wells, Alexandra Tsigarida, Beatriz Bezerra

**Affiliations:** ^1^ Department of Periodontology, Eastman Institute for Oral Health University of Rochester Rochester New York USA; ^2^ Statistical Methods and Data Analytics UCLA Office of Advanced Research Computing Los Angeles California USA; ^3^ Section of Periodontics, Division of Regenerative and Reconstructive Sciences UCLA School of Dentistry Los Angeles California USA

**Keywords:** biofilm, decontamination, dental implants, periimplantitis

## Abstract

**Objective:**

To assess which decontamination method(s) used for the debridement of titanium surfaces (disks and dental implants) contaminated with bacterial, most efficiently eliminate bacterial biofilms.

**Material and Methods:**

A systematic search was conducted in four electronic databases between January 1, 2010 and October 31, 2022. The search strategy followed the PICOS format and included only in vitro studies completed on either dental implant or titanium disk samples. The assessed outcome variable consisted of the most effective method(s)—chemical or mechanical— removing bacterial biofilm from titanium surfaces. A meta‐analysis was conducted, and data was summarized through single‐ and multi‐level random effects model (*p* < .05).

**Results:**

The initial search resulted in 5260 articles after the removal of duplicates. After assessment by title, abstract, and full‐text review, a total of 13 articles met the inclusion criteria for this review. Different decontamination methods were assessed, including both mechanical and chemical, with the most common method across studies being chlorhexidine (CHX). Significant heterogeneity was noted across the included studies. The meta‐analyses only identified a significant difference in biofilm reduction when CHX treatment was compared against PBS. The remaining comparisons did not identify significant differences between the various decontamination methods.

**Conclusions:**

The present results do not demonstrate that one method of decontamination is superior in eliminating bacterial biofilm from titanium disk and implant surfaces.

## 1 INTRODUCTION

Dental implants have become widely used to treat tooth loss. A study by Elani et al. (Elani et al., 2018) has projected that dental implant prevalence in the United States could be as high as 23% by 2026, with higher prevalence seen among patients with certain demographic characteristics, such as greater than high school degree and private insurance. With the increased numbers of implants being placed clinicians should be very diligent in diagnosing peri‐implant diseases at their early stages given the successful treatment of peri‐implantitis still presents its challenges. Peri‐implantitis is a plaque‐induced inflammatory condition developing around dental implants, which leads to loss of bone support with eventual implant failure (Berglundh et al., 2018). Its prevalence has been estimated to be 22% (Derks & Tomasi, 2015). Similar to periodontitis, the bacterial biofilm triggers a local inflammatory response, leading to initial signs of bleeding and/or suppuration upon probing, with progression to loss of supporting bone of the implant (Berglundh et al., 2018). The successful treatment of this condition is highly dependent upon the removal of the bacterial biofilm from the implant surface (Lindhe & Meyle, 2008).

Management of peri‐implantitis has proven quite challenging, in part due to our inability to adequately decontaminate the implant surface. This step in treatment is of primary importance for the successful resolution of the bony defects created by the disease (Persson et al., 1999). However, surgical treatment is often advocated for the management of peri‐implantitis as it allows the surgeon better access to the implant surface for decontamination and, an attempt to repair/recontour the osseous defects (Karring et al., 2005). Chemical decontamination methods are typically performed with agents that act upon the microbial biofilm in differing manners to either kill the bacterial populations present, reduce the replication of existing species, and/or modulate the local environment (Monje, Amerio, et al., 2022). Mechanical decontamination is performed via different methods, including the use of curettes, ultrasonic devices, or air‐abrasive powder systems among others reported in the literature (Monje et al., 2022).

Currently, no gold standard treatment for managing peri‐implantitis exists. This inconsistency is in part, due to the differing micro‐ and macro‐topographies of dental implant surfaces. Many studies agree that this complex geometry further complicates the complete removal of the bacterial biofilm from any exposed implant surface (Koo et al., 2019; Meyle, 2012). Current studies investigating the efficacy of decontamination methods have utilized titanium disks with micro‐topography mimicking that of dental implants as study samples. Given that the macrostructure of dental implants may play a role, several investigators have begun conducting studies on dental implant replicas to better translate to clinical practice (Azizi et al., 2018; Karimi et al., 2021; Patianna et al., 2018; Saffarpour et al., 2016).

The purpose of this systematic review and meta‐analysis is to assess which current decontamination method used for the debridement of titanium surfaces contaminated with bacterial biofilm, represented here by titanium disks and dental implants, most efficiently eliminates bacterial biofilm.

## 2 MATERIALS AND METHODS

### 2.1 Study design

A systematic review of in vitro studies examining the effects of different decontamination methods on the elimination of biofilm accumulated on titanium implant and titanium disk surfaces.

### 2.2 Reporting format

The reporting of this systematic review was guided by the standards of the Preferred Reporting Items for Systematic Review and Meta‐Analysis (PRISMA) Statement (Page et al., 2021).

### 2.3 Focused question

Which decontamination method most effectively removes bacterial biofilm from dental implant surfaces in vitro?

### 2.4 Population, intervention, comparison, outcome, study (PICOS) question

The focused question for the present study followed the following PICOS format:

Population (P): titanium surfaces (implants/titanium disks) contaminated with bacterial biofilm

Intervention, Comparison (I,C): decontamination method(s) (mechanical, chemical)

Outcome (O): bacterial biofilm removal from the affected surfaces

Study type (S): in vitro studies

### 2.5 Eligibility criteria

1. Inclusion criteria

a. Only in vitro studies were considered
b. Studies using dental implants and titanium disks
c. At least five samples per treatment group
d. Surfaces had to be contaminated with bacteria in vitro
e. Decontamination of sample completed on bench‐top
f. Studies published between January 1, 2010 and October 31, 2022
g. Published in English
h. Results reporting data on colony forming units (CFU)

2. Exclusion Criteria

a. Animal and *ex vivo* studies
b. Pilot studies
c. Studies on explanted implants
d. Less than five samples per treatment group
e. Surfaces not contaminated with bacteria in vitro
f. Decontamination of sample not completed on bench‐top
g. Studies published before January 1, 2010
h. Inability to obtain full text
i. No email response to inquiry email to corresponding authors
j. Published in other languages
k. Results not reporting data in CFU

### 2.6 Search strategy

Electronic and manual searches were conducted to identify studies reporting on different titanium surface decontamination methods. Four electronic databases were searched: MEDLINE, EMBASE, Cochrane, and Web of Science. In addition, the following publications were hand‐searched for relevant articles: Journal of Clinical Periodontology, Journal of Periodontology, Journal of Periodontal Research, Journal of Oral and Maxillofacial Surgery, and Clinical Oral Implant Research. The search was performed from January 1, 2010 to October 31, 2022. The search strategy used in all databases included the following descriptors and MeSH terms: (dental implant OR titanium disk OR titanium disc) AND (decontamination OR disinfection OR cleaning OR debridement) AND (biofilm reduction OR biofilm removal OR biofilm ablation).

### 2.7 Study selection

Articles were collected in reference manager software (EndNote, Thomson Reuters) and duplicates were discarded electronically. Titles and abstracts were screened by two calibrated reviewers (BB, IH) for potential inclusion. All titles and abstracts selected by the two reviewers were discussed individually for full‐text reading inclusion. If the title and abstract did not provide sufficient information regarding the inclusion criteria, the full text was reviewed. Full‐text reading of the selected publications was carried out independently by the reviewers. Consensus was reached at every step of the review. When disagreement between the two reviewers occurred, consensus was achieved by discussion with a third reviewer (AT). In cases where information was not clear or insufficient, the authors of the pertinent study were contacted by email to obtain further information.

### 2.8 Data extraction

Data collection was done using an electronic spreadsheet. Data were independently extracted and inserted into a computer by two calibrated reviewers (BB, IH) using specifically designed data‐collection forms. Data collected included: decontamination method, sample type (implant, titanium disk), sample surface type, sample size, bacterial species used for contamination, length of biofilm exposure, length of decontamination treatment, mean log CFU, and standard deviation posttreatment.

### 2.9 Quality assessment of included studies

Two reviewers (BB and IH) assessed the methodological quality of the studies included in the analysis. The assessment was completed using the QUIN tool (Sheth et al., 2022), which provides a standardized approach for evaluating the risk of bias of in vitro studies in Dentistry. This tool consists of 12 criteria, is simple to use, and allows for comparison between studies as it provides a quantitative assessment of risk. Studies with a score above 70% were considered to have low risk; scores between 50% and 70% were considered to have medium risk, and those below 50% were considered to have a high risk of bias.

### 2.10 Statistical analysis

The data of individual studies were pooled quantitatively to perform meta‐analysis using Stata statistical software (version 18; College Station, TX). Random effects (using REML) models were used when studies were not repeated in a data set; random effects (using REML) multilevel meta‐analysis was used when a study appeared more than once in a given data set. These methods were used to analyze the effects of different decontamination methods on the log CFU on both titanium disks and dental implant samples. The level of significance was set at 5%. Effect sizes were calculated in the metric Hedges' *g* for each comparison.

## 3 RESULTS

### 3.1 Study selection

**Figure 1:**
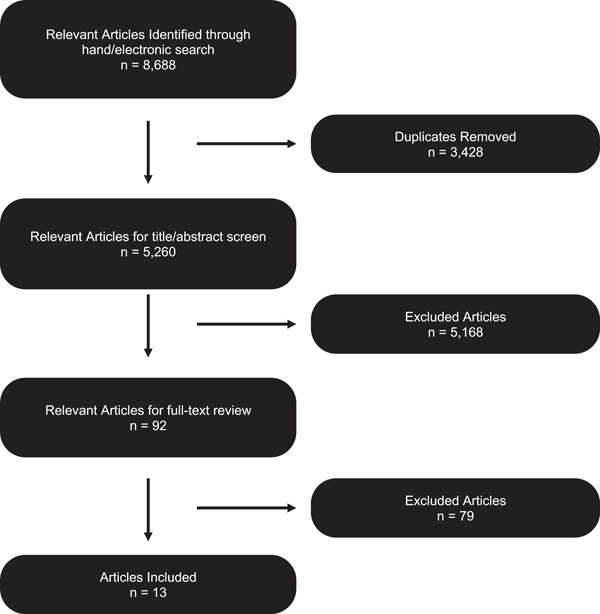
Flowchart of search and selection process.

A total of 5260 records were identified through search electronic databases and once duplicates had been removed (Figure 1). Five additional articles were included from other sources including hand‐searching relevant journals. After the screening of titles and abstracts, 5168 articles were removed, and the examiners reached accordance of 99% with a kappa score of 0.96 (Landis & Koch, 1977).

Ninety‐two articles were then retrieved, and the full texts were reviewed. The authors were contacted for the necessary data, had the data not been in the original articles. A further 79 articles were removed as they did not meet the inclusion criteria or there was no reply, leaving 13 articles that met the criteria for inclusion. Details of the included studies are summarized in Table 1. Table S1 lists data extracted for meta‐analysis. Table S2 lists excluded full‐text articles and reasons for exclusion.

**Table 1:** Details of studies included in a systematic review.

Author (year)	Sample type	Contamination	Treatment	Conclusion
Abushahba et al. (2021)	titanium disk	*P. gingivalis*	Zn4 Bioglass	Zn4 Bioglass and 45S5 Bioglass resulted in the complete elimination of *P. gingivalis* from the surfaces of titanium disks. *F. nucleatum* CFUs were significantly reduced with both bioglass formulations but not eliminated.
		*F. nucleatum*	45S5 Bioglass	
			Inert Glass	
			None	
Azizi et al. (2018)	implant	*A. actinomycetemcomitans*	0.2% CHX	Treatment of implant surfaces with PDT resulted in a significant reduction of bacteria from implant surfaces compared to other decontamination methods.
		*P. gingivalis*	PDT	
		*P. intermedia*	LED+Toluidine Blue	
			Toluidine Blue	
Cai, Li, Wang, Chen, Jiang, Ge, Lei, Huang (2019)	titanium disk	*S. aureus*	PBS	PDT in combination with either CHX or H2O2 was more effective in reducing the concentration of *S. aureus* on titanium disks than each treatment alone.
			0.2% CHX	
			3% H2O2	
			PDT	
			CHX + PDT	
			H2O2 + PDT	
Cho et al. (2015)	titanium disk	*A. actinomycetemcomitans*	PBS + Glass Beads	Treatment of titanium disks with Erythrosine and LED resulted in a significant reduction of bacterial counts compared to photosensitizer agent alone and control treatment.
			Erythrosine	
			Erythrosine LED	
Eick et al. (2013)	titanium disk	Multispecies	No Treatment	PDT alone or in combination with H2O2 resulted in a significant reduction of viable bacteria on the titanium disk surfaces.
			PDT	
			PDT + 0.25% H2O2	
Etemadi et al. (2020)	titanium disk	*A. actinomycetemcomitans*	PBS	CHX treatment of titanium disks resulted in the complete elimination of *A.a* from the treated surfaces.
			0.2% CHX	
			Phycocyanin	
			Diode Laser (DL)	
			Phycocyanin + DL	
Ghasemi et al. (2019)	titanium disk	*A. actinomycetemcomitans*	PBS	Treatment with CHX resulted in almost complete elimination of *A.a* from titanium disk surfaces.
			0.2% CHX	
			PDT	
			LED+ Toluidine Blue	
			Toluidine Blue	
			Sterile	
Namour et al. (2021)	titanium disk	multispecies biofilm	Nd:YAG	Nd:YAG laser treatment resulted in the complete elimination of bacterial biofilm from titanium disk surfaces.
			No Treatment	
			Sterile	
Ntrouka et al. (2011)	titanium disk	*S. mutans*	Sterile Water	Citric acid treatment of titanium disks resulted in the greatest bactericidal effect against *S. mutans*. Citric acid treatment was also significantly effective in eliminating biofilm cells from the disk surfaces.
			0.2% CHX	
			10% H2O2	
			Ardox‐X	
			Cetylpyridium Chloride	
			Citric Acid (40%)	
			EDTA (24%)	
Patianna et al. (2018)	implant	*S. sanguinis*	14% Doxycycline Gel	Application of a 14% doxycycline gel to implant surfaces for 3 min resulted in a significant reduction of *S. sanguinis* counts.
			Sterile Saline	
Karimi et al. (2021)	implant	*S. aureus*	Ti Brush	CHX treatment resulted in the greatest reduction of *S. aureus* from contaminated implant surfaces. The combination of titanium brush and citric acid showed similar results to CHX (*p* > .99).
			40% Citric Acid (CA)	
			Ti Brush + CA	
			Diode Laser	
			Ti Brush + Diode Laser	
			0.2% CHX	
			PBS	
			No Treatment	
Saffarpour et al. (2016)	implant	*A. actinomycetemcomitans*	Sterile Saline	Treatment of implant surfaces with CHX resulted in the greatest reduction of *A.a*. counts among all treatments, however, complete elimination of *A.a*. was not achieved by any of the treatments.
			2% CHX	
			Er:YAG	
			PDT	
			PDT	
Tonon et al. (2020)	titanium disk	*P. gingivalis*	Saline	80 micrograms/Nml Ozonized saline applied to titanium disks surfaces for 1 min resulted in similar bacterial counts compared to CHX.
		*F. nucleatum*	Ozonized Saline in various concentrations	
		*S. oralis*	0.12% CHX	

Abbreviations: CHX, chlorhexidine; H2O2, hydrogen peroxide; LED, laser emitting diode; PBS, phosphate‐buffered solution; PDT, photodynamic Therapy; Ti, titanium.

### 3.2 Quality assessment

Quality assessment of the included studies is presented in Table 2. The estimated risk of bias for four studies (Eick et al., 2013; Ntrouka et al., 2011; Patianna et al., 2018; Tonon et al., 2020) was high; the remaining studies (Alagl et al., 2019; Azizi et al., 2018; Cai, Li, Wang, Chen, Jiang, Ge, Lei, Huang, 2019; Cho et al., 2015; Etemadi et al., 2020; Ghasemi et al., 2019; Karimi et al., 2021; Namour et al., 2021; Saffarpour et al., 2016) presented a medium risk of bias. The included in vitro studies did not present detailed information regarding sample size calculation, randomization methods, and operator details among other aspects considered in the QUIN tool.

**Table 2:** Quality assessment of selected studies

Author	Alagl et al. (2019)	Azizi et al. (2018)	Cai, Li, Wang, Chen, Jiang, Ge, Lei, Huang (2019)	Cho et al. (2015)	Eick et al. (2013)	Etemadi et al. (2020)	Ghasemi et al. (2019)	Karimi et al. (2021)	Namour et al. (2021)	Ntrouka et al. (2011)	Patianna et al. (2018)	Saffarpour et al. (2016)	Toma et al. (2018)
**Quality Criteria**													
Aim/objectives	2	2	2	2	2	2	2	2	2	1	2	2	2
Sample size calculation	0	0	0	0	0	0	0	0	0	0	0	0	0
Sampling technique	1	1	2	1	1	1	1	0	0	1	1	1	1
Detail comparison groups	1	2	2	2	1	2	2	2	2	2	2	2	2
Detailed methodology	2	1	1	2	1	2	2	2	2	1	1	2	1
Operator details	1	1	0	1	1	0	0	1	0	0	0	1	0
Randomization	0	1	1	1	0	1	0	1	0	0	0	0	0
Methods of measurement of outcomes	1	1	1	1	0	1	1	1	1	2	1	0	1
Outcome assessor details	1	0	0	0	0	0	0	1	1	0	0	1	0
Blinding	2	0	0	0	0	0	0	1	0	0	0	1	0
Statistical analysis	2	2	2	2	1	2	2	0	2	2	2	2	2
Results	1	1	1	2	1	1	2	2	2	2	1	2	2
Final Score	58.3	50	50	58.3	33.3	50	50	54.2	50	45.8	41.7	58.3	45.8
**Estimated Risk of Bias**	MEDIUM	MEDIUM	MEDIUM	MEDIUM	HIGH	MEDIUM	MEDIUM	MEDIUM	MEDIUM	HIGH	HIGH	MEDIUM	HIGH

### 3.3 Surface decontamination

The included studies consisted of nine articles using titanium disks (Abushahba et al., 2021; Cai, Li, Wang, Chen, Jiang, Ge, Lei, Huang, 2019; Cho et al., 2015; Eick et al., 2013; Etemadi et al., 2020; Ghasemi et al., 2019; Namour et al., 2021; Ntrouka et al., 2011; Tonon et al., 2020), and four studies (Azizi et al., 2018; Karimi et al., 2021; Patianna et al., 2018; Saffarpour et al., 2016) using dental implants. Given the large heterogeneity present in the selected studies, the data from dental implant samples and titanium disks were combined for the meta‐analysis. All included studies utilized several different methods for decontamination, including both chemical and mechanical means, to clean titanium disks/implants with a sand‐blasted acid‐etched (SLA) surface. The most common method used for decontamination across all studies was chlorhexidine (CHX) either at a concentration of 0.2% or 0.12%. Other chemical approaches used hydrogen peroxide (H_2_O_2_), citric acid, EDTA, and cetylpyridium chloride. Physical approaches utilized air particle abrasion with different powders, titanium brushes, or laser treatment. Photodynamic therapy was a separate mode of therapy that was tested by multiple authors utilizing different photosensitizers.

A total of 10 articles were available for comparison (Alagl et al., 2019; Azizi et al., 2018; Cai, Li, Wang, Chen, Jiang, Ge, Lei, Huang, 2019; Cho et al., 2015; Etemadi et al., 2020; Ghasemi et al., 2019; Karimi et al., 2021; Ntrouka et al., 2011; Saffarpour et al., 2016; Tonon et al., 2020). Due to significant variation in the included studies, only certain comparisons were able to be completed through meta‐analysis.

#### 3.3.1 PBS versus CHX

A multilevel meta‐analysis was performed for this comparison as Tonon et al. (Tonon et al., 2020) contributed with more than one entry. Based upon the results of the meta‐analysis, CHX treatment of the samples had a larger effect in reducing the bacterial load as compared to treatment with saline alone (Hedge's g = 1.73, 95% CI [0.92, 2.55], *p* < .05, I^2^ = 68.98%). This difference, although significant, must be interpreted with caution as there is significant heterogeneity that could influence the true effect of the result.

#### 3.3.2 PBS versus H_2_O_2_

**Figure 2:**
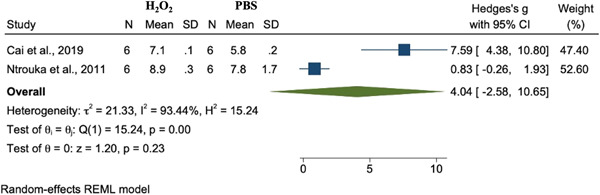
Meta‐analysis for the comparison between PBS and H_2_O_2_ decontamination methods.

The results of the meta‐analysis failed to show a significant difference in terms of decontamination when comparing the effects of saline alone as compared to treatment with hydrogen peroxide (Hedges' g = 4.04, 95% CI [−2.58, 10.65], *p* = .23, I^2^ = 93.44%) (Figure 2).

#### 3.3.3 PBS versus PDT

A multilevel meta‐analysis was performed for this comparison as three studies contributed with more than one entry (Cho et al., 2015; Ghasemi et al., 2019; Saffarpour et al., 2016). The results of the meta‐analysis failed to show a significant difference in terms of decontamination when comparing the effects of saline alone as compared to treatment via photodynamic therapy (Hedges' g = 4.16, 95% CI [−1.703,10.02], *p* = .0631, I^2^ = 99.09%).

#### 3.3.4 PBS versus photosensitizing agent alone

**Figure 3:**
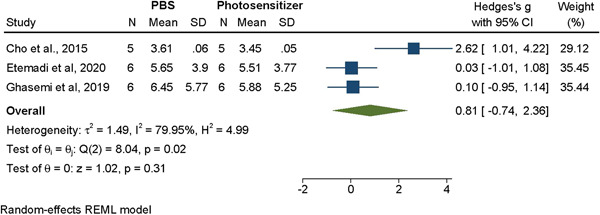
Meta‐analysis for the comparison between PBS and photosensitizing agent decontamination methods.

The results of the meta‐analysis failed to show a significant difference in terms of decontamination when comparing the effects of saline alone as compared to treatment with a photosensitized alone (Hedges' g = 0.18, 95% CI [−0.74, 2.36], *p* = .31, I^2^ = 79.95%) (Figure 3).

#### 3.3.5 PBS versus LASER

**Figure 4:**
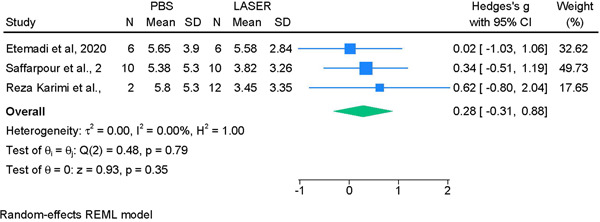
Meta‐analysis for the comparison between PBS and LASER decontamination methods.

No significant differences were noted between decontamination methods (*p* < .79). The average effect size (Hedge's g = 0.28, 95% CI [−0.31 to 0.88]) was small and not significant; and the test of heterogeneity was also not statistically significant (I^2^ = 0%) (Figure 4).

#### 3.3.6 CHX versus H_2_O_2_

**Figure 5:**
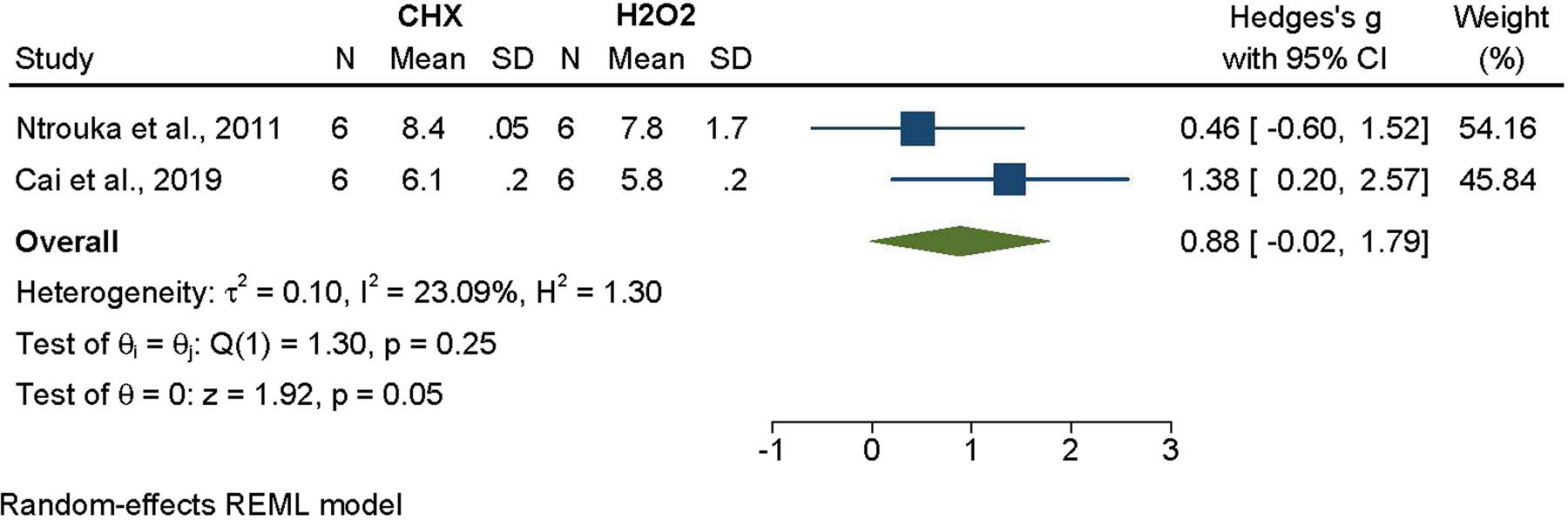
Meta‐analysis for the comparison between chlorhexidine and H_2_O_2_ decontamination methods.

The results found that there was a tendency toward a better disinfectant effect of CHX. However, the comparison was only between two studies, and the results of the meta‐analysis failed to show a significant difference in terms of decontamination when comparing the effects of CHX treatment as compared to treatment with H_2_O_2_ (Hedges' g = 0.88, 95% CI [−0.02, 1.79], *p* = .05, I^2^ = 23.09%) (Figure 5).

#### 3.3.7 CHX versus PDT

A multilevel meta‐analysis was also performed for this comparison as three studies contributed with more than one entry (Alagl et al., 2019; Azizi et al., 2018; Ghasemi et al., 2019). The results of the meta‐analysis failed to show a significant difference in terms of decontamination when comparing the effects of CHX treatment as compared to treatment with photodynamic therapy (Hedges' g = 0.73, 95% CI [−1.29 to 2.76], *p* = .00, I^2^ = 95.32%).

#### 3.3.8 CHX versus laser

A multilevel meta‐analysis was performed for this comparison as Alagl et al. (Alagl et al., 2019) had more than one entry included in the analysis. The results of the meta‐analysis failed to show a significant difference in terms of decontamination when comparing the effects of CHX treatment as compared to laser decontamination (Hedges' g = 0.04, 95% CI [−0.55 to 0.64], *p* = .03, I^2^ = 33.41%).

#### 3.3.9 CHX versus photosensitizing agent

A multilevel meta‐analysis was also performed for this comparison as two studies had more than one entry included in the analysis (Alagl et al., 2019; Azizi et al., 2018). Results did not show differences between the decontamination effects of chlorhexidine over the photosensitizing agents applied in the studies. The average effect size is small and not statistically significant (Hedges' g = −0.37, 95% CI [−1 to 0.257], *p* = .52, I^2^ = 42.25%).

#### 3.3.10 Laser versus PDT

A multilevel meta‐analysis was also performed for this comparison as two studies had more than one entry included in the analysis (Alagl et al., 2019; Saffarpour et al., 2016). The results of the meta‐analysis failed to show a significant difference in terms of decontamination when comparing the effects of photodynamic therapy treatment as compared to laser decontamination (Hedges' g =−0.25, 95% CI [−0.865 to 0.3347], *p* = .09, I^2^ = 33.84%).

## 4 DISCUSSION

Peri‐implant mucositis and peri‐implantitis are both biofilm‐induced conditions and as such, one of the most important aspects of treatment has been the decontamination of the implant surface (Berglundh et al., 2018). Several decontamination methods have been proposed and tested to remove bacterial biofilm from the implant surfaces, with the end goal of leaving the exposed surfaces amenable to recolonization by host cells. The methods included in the current systematic review, as well as other reviews, can commonly be classified as mechanical, chemical, or other (Monje et al., 2022). To our knowledge, no consensus has been reached regarding which methodology or combination of methodologies produces the most optimal result. No other systematic review has separately looked at the effects on titanium samples of different configurations (i.e. disc vs implant geometry).

Mechanical, chemical, and laser‐based strategies (laser treatment and photodynamic therapy) were all tested in the included studies. None of the studies utilizing mechanical means were able to be included in the meta‐analysis given significant differences in methodology (Abushahba et al., 2021; Cho et al., 2015). However, in these studies, significant reductions in biofilms were seen when air abrasion was completed with either differing formulations of bioglass (Abushahba et al., 2021) or erythrosine powder (Cho et al., 2015). Mechanical decontamination of the implant surface aims to remove bacterial biofilm while mitigating any significant change in the biocompatibility of the implant surface (Louropoulou et al., 2012, 2014). Implantoplasty, or the mechanical alteration of the implant surface to aid in hygiene practices, was not tested in any of the included studies. This was likely due to the inclusion of strictly in vitro studies. Current evidence supports implantoplasty as an effective therapy, however, these data come from clinical studies using surrogate clinical markers or radiographic measures to show the stability of the treated implants (Bianchini et al., 2019; Monje et al., 2022; Romeo et al., 2005, 2007).

Although mechanical therapy alone has shown significant improvements, it is commonly completed in conjunction with laser therapies or chemical means of decontamination. Laser therapy alone (Nd:YAG) was shown to be effective in reducing biofilm on the surface of SLA disks by Namour et al. (Namour et al., 2021). Interestingly, in this study, there was a complete eradication of the microbial population present on the disks, a rare finding among studies reporting bacterial reduction (Namour et al., 2021).

The two most common lasers that have been studied are the erbium‐based lasers (Er:YAG, Er:YSGG) and the diode lasers. Erbium‐based lasers are absorbed primarily by hydroxyapatite and water; therefore, they tend to be reflected by the implant surface. This is advantageous as the laser energy is not transferred to the implant, causing no damage to the implant surface nor an increase in the implant temperature, which is detrimental to the surrounding structures (Alagl et al., 2019; Kreisler et al., 2002; Romanos et al., 2017; Scarano et al., 2020; Strever et al., 2017). The efficacy of erbium‐based lasers in decontamination has been proven in previous studies, all supporting their use as an effective means for biofilm removal around implants of many surfaces and geometries (Alagl et al., 2019; Giannelli, Bani, et al., 2017; Linden et al., 2021; Takagi et al., 2018).

Diode lasers present wavelengths ranging from 810 to 980 nm, which are absorbed primarily by the surrounding tissues, and act to kill bacteria through thermal effects (Azma & Safavi, 2013). Similar to the erbium‐based lasers, multiple studies have found that diode lasers are effective in reducing the microbial population around dental implants while limiting the damage to the implant surface (Lollobrigida et al., 2020; Romanos et al., 2000; Sennhenn‐Kirchner et al., 2007; Tosun et al., 2012). However, the main concern with diode lasers is the potential for overheating the implant and causing irreversible damage to the surrounding vital structures (Eriksson & Albrektsson, 1983; Geminiani et al., 2012; Valente, Mang, et al., 2017). For these safety reasons, as well as evidence for non‐superior treatment outcomes to more traditional therapy, diode lasers have not been advocated for use in the decontamination of implant surfaces (Mattar et al., 2021). The use of other lasers (i.e., CO_2_ and Nd:YAG) has been documented and shows a strong bactericidal effect when utilized in low powers, without significantly altering the implant surface (Giannini et al., 2006; Kato et al., 1998; Namour et al., 2021). Their use, however, is of limited benefit as compared to conventional therapy utilizing either mechanical or chemical approaches (Monje et al., 2022).

Photodynamic therapy (PDT), either utilizing an LED light source or diode laser, was utilized by many of the included studies as a method for decontamination of titanium disks and implants (Azizi et al., 2018; Cai, Li, Wang, Chen, Jiang, Ge, Lei, Huang, 2019; Eick et al., 2013; Etemadi et al., 2020; Ghasemi et al., 2019; Saffarpour et al., 2016). These studies showed reductions in bacterial load when photodynamic therapy was used in comparison to their respective control groups. This supports the idea that the use of a photosensitizer, activated by light at a specific wavelength to produce reactive oxygen species (ROS), is an effective means of reducing the bacterial load on implants (Choe et al., 2021). The production of ROS causes damage to the bacterial cell membrane and presents cytotoxic effects on viruses, fungi, and protozoa (Takasaki et al., 2009). The advantage of PDT is that it does not rely on mechanical access to the area, rather the radius of activation is quite small, and thus it may have the ability to reach areas that would otherwise be near impossible to access. Moreover, since there is no need to directly contact the implant surface, PDT is safe and does not alter the implant surface (Alasqah, 2019). In the current meta‐analysis, PDT was not found to produce significantly greater results than the use of PBS/saline alone, however, it was also not found to produce significantly worse results than CHX; therefore, the true efficacy is still unknown.

Many of the included studies also utilized chemical approaches to decontaminate the surface of the titanium disks. Of these, CHX and H_2_O_2_ were the only chemical substances that were able to be included in the meta‐analysis. In our findings, CHX was more effective than PBS/saline at reducing the bacterial load on titanium disks and implants; however, when compared to all other methods of decontamination, CHX failed to show a statistically significant benefit. Chlorhexidine has been widely studied as a chemical decontaminant as it is one of the most widely used substances in periodontics. It acts through disruption of the cell membranes of bacteria causing cell death (Jenkins et al., 1988). While many studies agree that CHX is an effective agent for biofilm reduction on the implant surfaces, there has been recent concern surrounding its cytotoxicity to host cells, influencing the biocompatibility of the implant surface following treatment (Brunello et al., 2021; Cai, Li, Wang, Chen, Jiang, Ge, Lei, Huang, 2019; Etemadi et al., 2020; Ghasemi et al., 2019; Kotsakis et al., 2016; Ntrouka et al., 2011; Tonon et al., 2020). For this reason, some authors have suggested eliminating the frequent use of CHX as an agent for biofilm reduction.

Hydrogen peroxide, which acts on a broad range of bacteria through the production of reactive oxygen species, interacts and alters cellular components leading to cell death (Linley et al., 2012). Although the included studies that utilized H_2_O_2_ noted bacterial reduction, the results of the current meta‐analysis did not find H_2_O_2_ to be more efficacious than any other method of decontamination, including sterile saline, which is in agreement with other studies that have examined the use of H_2_O_2_ (Bürgers et al., 2012; Cai, Li, Wang, Chen, Jiang, Ge, Lei, Huang, 2019; Ntrouka et al., 2011) (Figures 2, 5).

Ntrouka et al. (Ntrouka et al., 2011) looked further at the antimicrobial effects of 24% ethylenediaminetetraacetic acid (EDTA), 40% citric acid (CA), 0.07% cetylpyridium chloride, and Ardox‐X®. Ardox‐X® is a proprietary compound that provides a controlled release of active oxygen without generating hydroxyl radicals. It acts as a matrix releasing active oxygen to the area being treated (Fernandez y Mostajo et al., 2014). In this study, CA and Ardox‐X® were found to be the most potent treatments in terms of CFU reduction (Ntrouka et al., 2011). Citric acid, a weak acid, has the ability to diffuse into the cell in its undissociated state, decreasing the intracellular pH and altering the structure and function of the cell (Burel et al., 2021). It has been proven to be an effective means to decontaminate implant surfaces with minimal disruption to the biocompatibility following treatment, making it a good agent for decontamination (Han et al., 2019; Kotsakis et al., 2016; Souza et al., 2018). EDTA is a chelating agent that sequesters metal ions from the outer membrane, potentially weakening it (Sen et al., 2000; Stojicic et al., 2012). EDTA's bactericidal effect is unknown, as some argue it has no effect, which is supported by Ntrouka et al. (Ntrouka et al., 2011) findings that it is no different than sterile water. Other studies, however, found positive effects when combining EDTA with other therapies (Kotsakis et al., 2016). Therefore, the use of EDTA shows promise and is recommended to preserve the biocompatibility of the implant and enhance the antimicrobial effects of other decontaminating agents (Monje et al., 2022). However, when used as a monotherapy, it may not provide adequate decontamination to achieve the desired outcomes (Monje et al., 2022).

Lastly, PBS/sterile saline is often the control group for many studies as it does not have specific chemical action. However, the mechanical removal of bacteria from the implant surface with the use of a saline‐soaked cotton pellet is beneficial as a decontamination method (Alhag et al., 2008; Kolonidis et al., 2003; Persson et al., 1999). Supportive authors believe that saline has many benefits as it does not affect the implant surface structure or biocompatibility and is also cheap and readily available (Brunello et al., 2021; Kotsakis et al., 2016; Monje et al., 2022). Although the current meta‐analysis and other in vitro studies show that PBS/saline tends to perform more poorly than other mechanical, laser, or chemical means, in vivo studies where saline was combined with other forms of decontamination showed promising results (Alhag et al., 2008; Kolonidis et al., 2003; Persson et al., 1999). For these reasons it is not advised to use saline alone, but rather in conjunction with other methods to aid with decontamination.

Overall, from the results of the meta‐analysis, it appears that most treatments, including the use of PBS/saline alone, produce a reduction in the biofilm to a certain degree. When compared, most methods failed to show any significant differences in terms of bacterial reduction. The only significant difference found was CHX compared to PBS/saline treatment (Figure 3). However, this assessment, like most of the others, had a very high level of heterogeneity in the data. This heterogeneity could be masking true differences in efficacy; it comes as a result of a lack of standardization in methodology across different studies. Thus, it is very difficult to control.

Unfortunately, given the limited number of studies that met the inclusion criteria, mechanical means of decontamination were not able to be compared to chemical methods. Some of the included studies though, did report this method as effective in reducing the bacterial load of contaminated surfaces (Abushahba et al., 2021; Namour et al., 2021; Saffarpour et al., 2016).

The use of a titanium brush was tested by Karimi et al. (Karimi et al., 2021) and shown to be an effective mechanical means to disrupt and remove biofilm from the implant surface. Other studies have found titanium brushes to be a useful method for removing mineralized deposits from the implant surfaces, although there is some concern over alterations of the implant surface (Gonzalez et al., 2021; Louropoulou et al., 2014; Sanz‐Martin et al., 2021). In clinical studies, the use of titanium brushes shows promising results, producing significant clinical improvement when they are incorporated into peri‐implantitis therapy (Jepsen et al., 2016; Roccuzzo et al., 2016).

The only other method that was used on implant samples that was not utilized on the disks was doxycycline gel (14%) (Patianna et al., 2018). Patianna et al. (2018) found significant reductions in the remaining CFUs present on implants treated with doxycycline gel in comparison to sterile saline. Antibiotic therapy, most notably with tetracycline‐based antibiotics, has been found to reduce bacterial counts and lead to positive short‐term clinical outcomes in vivo (Kotsakis et al., 2021; Ramos et al., 2018). The effects are due to the antibacterial activity of tetracyclines themselves, inhibiting the 30 S ribosomal subunit. Doxycycline should be used as an adjunct, and not as monotherapy, as removal of biofilm is necessary for its effects to be realized (Monje et al., 2022).

Dental implant and disc samples were combined in this study's analysis, as the authors believed that given the in vitro nature of the study design, the ability to remove bacterial biofilm from a flat surface (disk) versus a screw‐like feature (implant) would not present a significant impact as compared to decontamination in a clinical scenario. However, we do believe that the use of dental implant samples should be taken into consideration in future in vitro studies, as it will allow for a better understanding of the impact of various decontamination methods on the macro‐ and micro‐topography of these samples. The impact of mechanical and chemical decontamination protocols on the microtopography of dental implants can promote surface alterations that may be detrimental to the clinical outcomes of periimplantitis treatment if these alterations result in surfaces that are more prone to bacterial biofilm accumulation. This study presents several limitations including the small number of studies included in the final analysis and the inability to perform certain comparisons given the lack of common treatment groups between the included studies. Another limitation was the high heterogeneity of the data, a factor of different methodologies applied by different studies. The non‐standardized methods of contamination, where some studies exposed samples to single bacterium biofilms, whereas others used multi‐bacteria biofilm, as well as different contamination periods, may also account for the variable results in decontamination among studies. Decontamination methods also varied in the included studies, and even though some methods were similar, variations still existed regarding the concentration of chemical agents, length of treatment, and so forth.

## 5 CONCLUSION

From the present results, it cannot be concluded that one method of decontamination was significantly more effective than another. Rather, most of the available in vitro studies show that all methods are effective in decontaminating titanium surfaces to a certain degree and none completely removed all the bacteria from the sample. In vitro studies are important for identifying new treatment methodologies, however, variations in methodologies hinder the ability to systematically assess the results and determine which studied methods can be safely translated to the clinical environment. Also, tools for assessing the quality of in vitro studies in Dentistry that have been widely validated in the literature should be considered to provide the readers with a greater level of confidence that the results reported have a low level of bias.

## AUTHOR CONTRIBUTIONS

Iain Hart contributed to the study design, data collection, data interpretation, and drafting of the manuscript. Christine Wells contributed to data analysis, data interpretation, and drafting of the manuscript. Alexandra Tsigarida contributed to the study design, data collection, and drafting of the manuscript. Beatriz Bezerra contributed to the study conception, design, data collection, data interpretation, and drafting of the manuscript.

## CONFLICT OF INTEREST STATEMENT

The authors declare no conflict of interest.

## Supporting information

Supporting information.

## Data Availability

The data that support the findings of this study are available from the corresponding author upon reasonable request.
