# Supplementary material for: Effectiveness of mechanical and chemical decontamination methods for the treatment of dental implant surfaces affected by peri‐implantitis: A systematic review and meta‐analysis
Source: Clin Exp Dent Res. 2024 Feb 6;10(1):e839. doi: 10.1002/cre2.839 (PMC10847712; doi:10.1002/cre2.839)
Supplement: Supplementary file 1 — Supporting information. [file CRE2-10-e839-s001.docx]

Supplemental Table 1. Data extracted for Meta-analysis.

| **Study** | **Decontamination Method** | **Surface Type** | **Sample Size** | **Bacterial Species** | **Biofilm Exposure (hours)** | **Length of Treatment (seconds)** | **Log CFU (mean)** | **Log CFU (SD)** |
| --- | --- | --- | --- | --- | --- | --- | --- | --- |
| **Abushahba et al., 2021** | Zn4 Bioglass | SLA | 5 | Multispecies | 70 | 20 | 4.24 | 3.81 |
|  | 45S5 Bioglass |  | 5 |  |  | 20 | 4.44 | 3.98 |
|  | Inert Glass |  | 5 |  |  | 20 | 6.52 | 6.23 |
|  | None |  | 5 |  |  | 0 | 7.06 | 6.82 |
|  |  |  |  |  |  |  |  |  |
| **Azizi et al., 2018** | 0.2% CHX | SLA | 12 | *A. actinomycetemcomitans* | 72 | 60 | 7.64 | 7.93 |
|  | 0.2% CHX |  | 12 | *P. gingivalis* |  | 60 | 7.22 | 7.50 |
|  | 0.2% CHX |  | 12 | *P. intermedia* |  | 60 | 7.87 | 8.08 |
|  | Negative Control |  | 12 | *A. actinomycetemcomitans* |  | 60 | 8.08 | 8.46 |
|  | Negative Control |  | 12 | *P. gingivalis* |  | 60 | 8.51 | 8.83 |
|  | Negative Control |  | 12 | *P. intermedia* |  | 60 | 8.53 | 8.74 |
|  | PDT (155ug/mL Toluidine Blue) |  | 12 | *A. actinomycetemcomitans* |  | 120 | 5.31 | 5.52 |
|  | PDT (155ug/mL Toluidine Blue) |  | 12 | *P. gingivalis* |  | 120 | 6.82 | 7.30 |
|  | PDT (155ug/mL Toluidine Blue) |  | 12 | *P. intermedia* |  | 120 | 6.82 | 7.09 |
|  | PDT (10mg/mL Phenothiazine Chloride) |  | 12 | *A. actinomycetemcomitans* |  | 120 | 5.28 | 5.75 |
|  | PDT (10mg/mL Phenothiazine Chloride) |  | 12 | *P. gingivalis* |  | 120 | 5.40 | 5.76 |
|  | PDT (10mg/mL Phenothiazine Chloride) |  | 12 | *P. intermedia* |  | 120 | 7.22 | 7.76 |
|  | LED (1mg/mL Toluidine Blue) |  | 12 | *A. actinomycetemcomitans* |  | 120 | 7.78 | 8.23 |
|  | LED (1mg/mL Toluidine Blue) |  | 12 | *P. gingivalis* |  | 120 | 7.45 | 7.78 |
|  | LED (1mg/mL Toluidine Blue) |  | 12 | *P. intermedia* |  | 120 | 8.20 | 8.42 |
|  | 1mg/mL Toluidine Blue |  | 12 | *A. actinomycetemcomitans* |  | 60 | 7.92 | 8.07 |
|  | 1mg/mL Toluidine Blue |  | 12 | *P. gingivalis* |  | 60 | 7.53 | 7.63 |
|  | 1mg/mL Toluidine Blue |  | 12 | *P. intermedia* |  | 60 | 8.25 | 8.41 |
|  |  |  |  |  |  |  |  |  |
| **Cai et al., 2019** | PBS | SLA | 6 | *S.aureus* | 48 | 60 | 7.10 | 0.10 |
|  | 0.2% CHX |  | 6 |  |  | 60 | 6.10 | 0.20 |
|  | 3% H2O2 |  | 6 |  |  | 60 | 5.80 | 0.20 |
|  | PDT+toluidine blue |  | 6 |  |  | 120 | 5.10 | 0.10 |
|  | CHX+PDT |  | 6 |  |  | 120 | 2.90 | 0.30 |
|  | H2O2+PDT |  | 6 |  |  | 120 | 3.30 | 0.40 |
|  |  |  |  |  |  |  |  |  |
| **Cho et al., 2015** | PBS + Glass Beads | SLA | 5 | *A. actinomycetemcomitans* | 72 | 60 | 3.61 | 0.06 |
|  | Erythrosine |  | 5 |  |  | 60 | 3.45 | 0.05 |
|  | Erythrosine 30 LED |  | 5 |  |  | 90 | 2.62 | 0.22 |
|  | Erythrosine 60 LED |  | 5 |  |  | 120 | 2.59 | 0.27 |
|  |  |  |  |  |  |  |  |  |
| **Eick et al., 2013** | No Treatment | SLA | 6 | *Multispecies* | 96 | 0 | 6.27 | 0.25 |
|  | PDT+toluidine blue |  | 6 |  |  | 60 | 5.41 | 0.10 |
|  | PDT+toluidine blue+0.25% H2O2 |  | 6 |  |  | 60 | 5.10 | 0.13 |
|  |  |  |  |  |  |  |  |  |
| **Etemadi et al., 2020** | PBS | SLA | 6 | *A. actinomycetemcomitans* | 48 | 0 | 5.65 | 3.90 |
|  | 0.2% CHX |  | 6 |  |  | 300 | 0.00 | 0.00 |
|  | Phycocyanin |  | 6 |  |  | 300 | 5.51 | 3.77 |
|  | Diode Laser |  | 6 |  |  | 240 | 5.58 | 2.84 |
|  | Phycocyanin + Diode Laser |  | 6 |  |  | 540 | 5.43 | 4.04 |
|  |  |  |  |  |  |  |  |  |
| **Ghasemi et al., 2019** | PBS | SLA | 6 | *A. actinomycetemcomitans* | 48 | 0 | 6.45 | 5.77 |
|  | 0.2% CHX |  | 6 |  |  | 300 | 1.96 | 2.06 |
|  | PDT+toluidine blue |  | 6 |  |  | 330 | 4.42 | 3.84 |
|  | LED+ Toluidine Blue |  | 6 |  |  | 330 | 3.96 | 3.38 |
|  | Toluidine Blue |  | 6 |  |  | 300 | 5.88 | 5.25 |
|  | Sterile |  | 6 | Clean |  | 0 |  |  |
|  |  |  |  |  |  |  |  |  |
| **Namour et al., 2021** | Nd:YAG | SLA | 24 | Multispecies | 48 | 2 | 0.00 | 0.00 |
|  | No Treatment |  | 24 |  |  | 0 | 3.67 | 3.28 |
|  | Sterile |  | 24 | Clean | 0 | 0 | 0.00 | 0.00 |
|  |  |  |  |  |  |  |  |  |
| **Ntrouka et al., 2011** | Sterile Water | SLA | 6 | *S. mutans* | 24 | 300 | 9.40 | 0.06 |
|  | 0.2% CHX |  | 6 |  |  | 300 | 8.10 | 0.48 |
|  | 10% H2O2 |  | 6 |  |  | 300 | 5.90 | 0.57 |
|  | Ardox-X |  | 6 |  |  | 300 | 4.20 | 0.84 |
|  | Cetylpyridium chloride |  | 6 |  |  | 300 | 8.70 | 0.09 |
|  | Citric Acid (CA) (40%) |  | 6 |  |  | 300 | 3.40 | 1.00 |
|  | EDTA (24%) |  | 6 |  |  | 300 | 9.10 | 0.16 |
|  |  |  |  |  |  |  |  |  |
| **Patianna et al., 2018** | 14% Doxycycline Gel | SLA | 10 | *S. sanguinis* | 24 | 180 | 2.01 | 2.28 |
|  | Sterile Saline |  | 11 |  |  | 60 | 2.74 | 3.13 |
|  |  |  |  |  |  |  |  |  |
| **Karimi et al., 2021** | Ti Brush | SLA | 12 | *S. aureus* | 72 | 120 | 3.65 | 3.29 |
|  | 40% Citric Acid (CA) |  | 12 |  |  | 120 | 3.60 | 3.68 |
|  | Ti Brush + CA |  | 12 |  |  | 240 | 2.78 | 2.54 |
|  | Diode Laser |  | 12 |  |  | 60 | 3.45 | 3.35 |
|  | Ti Brush + Diode Laser |  | 12 |  |  | 180 | 3.28 | 3.06 |
|  | 0.2% CHX |  | 12 |  |  | 60 | 2.38 | 2.64 |
|  | PBS |  | 2 |  |  | 60 | 5.81 | 5.31 |
|  | No Treatment |  | 1 | N/A | 0 | 0 | 0.00 | 0.00 |
|  |  |  |  |  |  |  |  |  |
| **Saffarpour et al., 2016** | Sterile Saline | SLA | 10 | *A. actinomycetemcomitans* | 48 | 30 | 5.38 | 41158.77 |
|  | 2% CHX |  | 10 |  |  | 30 | 3.04 | 5.90 |
|  | Er:YAG |  | 10 |  |  | 60 | 3.82 | 3.26 |
|  | PDT |  | 10 |  |  | 120 | 3.55 | 3.14 |
|  | PDT |  | 10 |  |  | 360 | 3.57 | 3.09 |
|  |  |  |  |  |  |  |  |  |
| **Tonon et al., 2020** | Sterile Physiologic Saline (negative control) | SLA | 9 | *P. gingivalis* | 120 | 60 | 5.53 | 0.49 |
|  | Ozonized Physiologic Saline [25μg/mL] |  | 9 |  |  | 30 | 4.83 | 0.32 |
|  | Ozonized Physiologic Saline [50μg/mL] |  | 9 |  |  | 30 | 3.97 | 0.83 |
|  | Ozonized Physiologic Saline [80μg/mL] |  | 9 |  |  | 30 | 2.75 | 1.66 |
|  | Ozonized Physiologic Saline [25μg/mL] |  | 9 |  |  | 60 | 3.59 | 2.16 |
|  | Ozonized Physiologic Saline [50μg/mL] |  | 9 |  |  | 60 | 4.43 | 0.55 |
|  | Ozonized Physiologic Saline [80μg/mL] |  | 9 |  |  | 60 | 3.66 | 1.52 |
|  | 0.12% CHX |  | 9 |  |  | 60 | 4.18 | 0.47 |
|  | Sterile Physiologic Saline (negative control) |  | 9 | *F. nucleatum* |  | 60 | 5.74 | 0.53 |
|  | Ozonized Physiologic Saline [25μg/mL] |  | 9 |  |  | 30 | 5.49 | 0.31 |
|  | Ozonized Physiologic Saline [50μg/mL] |  | 9 |  |  | 30 | 4.59 | 0.48 |
|  | Ozonized Physiologic Saline [80μg/mL] |  | 9 |  |  | 30 | 4.85 | 0.65 |
|  | Ozonized Physiologic Saline [25μg/mL] |  | 9 |  |  | 60 | 5.67 | 0.32 |
|  | Ozonized Physiologic Saline [50μg/mL] |  | 9 |  |  | 60 | 4.69 | 0.31 |
|  | Ozonized Physiologic Saline [80μg/mL] |  | 9 |  |  | 60 | 4.76 | 0.51 |
|  | 0.12% CHX |  | 9 |  |  | 60 | 4.85 | 0.36 |
|  | Sterile Physiologic Saline (negative control) |  | 9 | *S. oralis* |  | 60 | 6.24 | 0.72 |
|  | Ozonized Physiologic Saline [25μg/mL] |  | 9 |  |  | 30 | 5.66 | 0.76 |
|  | Ozonized Physiologic Saline [50μg/mL] |  | 9 |  |  | 30 | 5.97 | 0.34 |
|  | Ozonized Physiologic Saline [80μg/mL] |  | 9 |  |  | 30 | 6.19 | 0.49 |
|  | Ozonized Physiologic Saline [25μg/mL] |  | 9 |  |  | 60 | 5.82 | 0.94 |
|  | Ozonized Physiologic Saline [50μg/mL] |  | 9 |  |  | 60 | 6.16 | 0.43 |
|  | Ozonized Physiologic Saline [80μg/mL] |  | 9 |  |  | 60 | 6.30 | 0.57 |
|  | 0.12% CHX |  | 9 |  |  | 60 | 5.68 | 1.11 |
|  | Sterile Physiologic Saline (negative control) |  | 9 | Multispecies (Pg) |  | 60 | 5.42 | 0.45 |
|  | Sterile Physiologic Saline (negative control) |  | 9 | Multispecies (Fn) |  | 60 | 5.76 | 0.05 |
|  | Sterile Physiologic Saline (negative control) |  | 9 | Multispecies (So) |  | 60 | 6.61 | 0.04 |
|  | Ozonized Physiologic Saline [25μg/mL] |  | 9 | Multispecies (Pg) |  | 30 | 4.83 | 0.32 |
|  | Ozonized Physiologic Saline [25μg/mL] |  | 9 | Multispecies (Fn) |  | 30 | 5.50 | 0.33 |
|  | Ozonized Physiologic Saline [25μg/mL] |  | 9 | Multispecies (So) |  | 30 | 6.33 | 0.29 |
|  | Ozonized Physiologic Saline [50μg/mL] |  | 9 | Multispecies (Pg) |  | 30 | 3.97 | 0.83 |
|  | Ozonized Physiologic Saline [50μg/mL] |  | 9 | Multispecies (Fn) |  | 30 | 4.62 | 0.45 |
|  | Ozonized Physiologic Saline [50μg/mL] |  | 9 | Multispecies (So) |  | 30 | 6.26 | 0.19 |
|  | Ozonized Physiologic Saline [80μg/mL] |  | 9 | Multispecies (Pg) |  | 30 | 3.62 | 0.61 |
|  | Ozonized Physiologic Saline [80μg/mL] |  | 9 | Multispecies (Fn) |  | 30 | 4.63 | 0.68 |
|  | Ozonized Physiologic Saline [80μg/mL] |  | 9 | Multispecies (So) |  | 30 | 6.28 | 0.33 |
|  | Ozonized Physiologic Saline [25μg/mL] |  | 9 | Multispecies (Pg) |  | 60 | 4.21 | 1.09 |
|  | Ozonized Physiologic Saline [25μg/mL] |  | 9 | Multispecies (Fn) |  | 60 | 5.55 | 0.48 |
|  | Ozonized Physiologic Saline [25μg/mL] |  | 9 | Multispecies (So) |  | 60 | 6.16 | 0.43 |
|  | Ozonized Physiologic Saline [50μg/mL] |  | 9 | Multispecies (Pg) |  | 60 | 4.12 | 0.59 |
|  | Ozonized Physiologic Saline [50μg/mL] |  | 9 | Multispecies (Fn) |  | 60 | 4.47 | 0.53 |
|  | Ozonized Physiologic Saline [50μg/mL] |  | 9 | Multispecies (So) |  | 60 | 6.20 | 0.33 |
|  | Ozonized Physiologic Saline [80μg/mL] |  | 9 | Multispecies (Pg) |  | 60 | 4.16 | 0.66 |
|  | Ozonized Physiologic Saline [80μg/mL] |  | 9 | Multispecies (Fn) |  | 60 | 4.53 | 0.50 |
|  | Ozonized Physiologic Saline [80μg/mL] |  | 9 | Multispecies (So) |  | 60 | 6.22 | 0.26 |
|  | 0.12% CHX |  | 9 | Multispecies (Pg) |  | 60 | 4.20 | 0.41 |
|  | 0.12% CHX |  | 9 | Multispecies (Fn) |  | 60 | 4.85 | 0.46 |
|  | 0.12% CHX |  | 9 | Multispecies (So) |  | 60 | 5.45 | 0.63 |
| PDT: photodynamic Therapy; PBS: phosphate-buffered solution; LED: Laser emitting diode; H2O2: hydrogen peroxide; CHX: chlorhexidine; Ti: titanium. | | | | | | | | |

Supplemental Table 2. Excluded studies and reasons for exclusion.

| **Author** | **Sample Type** | **Reason for Exclusion** |
| --- | --- | --- |
| Abushahba et al., 2019 | disk | no response from authors |
| Al-Hashedi A et al., 2017 | disk | no CFU data and no response from author |
| Almoharib H et al., 2021 | disk | no CFU data and no response from author |
| Amate-Fernandez et al., 2021 | disk | no CFU data and no response from author |
| Balderrama et al., 2021 | implant | pilot study |
| Barrak et al., 2020 | disk | less than 5 samples per group |
| Batsukh et al., 2017 | disk | did not assess decontamination |
| Bernardi et al., 2019 | disk | less than 5 samples per group |
| Birang et al., 2019 | disk | in vivo bacterial contamination |
| Burges et al., 2012 | disk | no CFU data and no response from author |
| Cai et al., 2019 | disk | no response from authors |
| Camacho-Alonso et al., 2021 | disk | no response from authors |
| Charalamoakis et al., 2015 | disk | in vivo bacterial contamination |
| Chen et al., 2016 A | disk | no CFU data and no response from author |
| Chen et al., 2016 B | disk | no CFU data and no response from author |
| Cochis et al., 2013 | disk | did not assess decontamination |
| Cordeiro et al., 2021 | disk | in vivo bacterial contamination |
| de Fatima Balderrama et al., 2020 | implant | pilot study |
| Di Salle et al., 2018 | disk | did not assess decontamination |
| Dostie et al., 2017 | disk | in vivo bacterial contamination |
| Drago et al., 2014 | disk | did not assess decontamination |
| Drago et al., 2017 | disk | did not assess decontamination |
| Eick et al., 2017 | disk | no response from authors |
| Faccioni et al., 2021 | disk | less than 5 samples per group |
| Ferreira et al., 2015 | disk | less than 5 samples per group |
| Ferreira et al., 2015 A | disk | less than 5 samples per group |
| Gianelli et al., 2016 | disk | less than 5 samples per group |
| Gianelli et al., 2017 | disk | less than 5 samples per group |
| Gianelli et al., 2017 A | disk | less than 5 samples per group |
| Gumus et al., 2020 | disk | in vivo bacterial contamination |
| Gustumhaugen et al., 2013 | disk | no CFU data and no response from author |
| Han et al., 2019 | disk | less than 5 samples per group |
| Hauser-Gerspach et al., 2014 | disk | no response from authors |
| Henderson et al., 2013 | disk | no CFU data and no response from author |
| Huang et al., 2019 | disk | no CFU data |
| Huang et al., 2019 A | disk | no response from authors |
| Huang et al., 2021 | disk | in vivo bacterial contamination |
| Janson et al., 2018 | disk | no CFU data and no response from author |
| Jin et al., 2019 | disk | no CFU data |
| John et al., 2014 | disk | no CFU data |
| John et al., 2016 | disk | no CFU data |
| Koopaie et al., 2020 | disk | no CFU data |
| Kotsakis et al., 2016 | disk | less than 5 samples per group |
| Kotsakis et al., 2020 | disk | no response from authors |
| Kubasiewicz-Ross et al., 2020 | implant | no CFU data |
| Kubasiewicz-Ross et al., 2020 A | implant | no CFU data |
| Larsen et al., 2017 | implant | no CFU data |
| Leelanarathiwat et al., 2020 | disk | no response from authors |
| Lollobrigida et al., 2019 | disk | no response from authors |
| Lubin et al., 2014 | disk | no CFU data |
| Mang et al., 2016 | disk | no response from authors |
| Marotti et al., 2013 | implant | in vivo bacterial contamination |
| Mensi et al., 2018 | disk | no response from authors |
| Meto et al., 2019 | disk | no response from authors |
| Nagao et al., 2019 | disk | less than 5 samples per group |
| Namour et al., 2020 | disk | no CFU data |
| Neimer-Vieira et al., 2012 | implant | no CFU data |
| Otsuki et al., 2020 | implant | in vivo bacterial contamination |
| Park et al., 2020 | disk | no CFU data |
| Passarelli et al., 2020 | implant | less than 5 samples per group |
| Quinteiro et al., 2017 | implant | decontamination not completed on bench-top |
| Ready et al., 2015 | disk | less than 5 samples per group |
| Sayar et al., 2019 | disk | no response from authors |
| Schmage et al., 2012 | disk | no CFU data |
| Schmage et al., 2014 | disk | no CFU data |
| Schuldt et al., 2021 | disk | no CFU data |
| Sharab et al., 2020 | disk | no response from authors |
| Souza et al., 2018 | disk | in vivo bacterial contamination |
| Strever et al., 2017 | disk | no CFU data |
| Stuani et al., 2021 | implant | less than 5 samples per group |
| Tastepe et al., 2013 | disk | no CFU data |
| Terlep et al., 2021 | disk | no CFU data |
| Toma et al., 2018 | disk | no CFU data |
| Tosun et al., 2012 | disk | less than 5 samples per group |
| Valente et al., 2017 | implant | decontamination not completed on bench-top |
| Venkei et al., 2020 | disk | no CFU data |
| Widodo et al., 2016 | disk | less than 5 samples per group |
| Wiedmer et al., 2017 | disk | no CFU data |
| Zoccolillo et al., 2016 | disk | no response from authors |
